# Ethanolic extracts of *Pluchea indica* (L.) leaf pretreatment attenuates cytokine-induced β-cell apoptosis in multiple low-dose streptozotocin-induced diabetic mice

**DOI:** 10.1371/journal.pone.0212133

**Published:** 2019-02-19

**Authors:** Jongdee Nopparat, Aekkaraj Nualla-ong, Amornrat Phongdara

**Affiliations:** 1 Department of Anatomy, Faculty of Science, Prince of Songkla University, Hat Yai, Songkhla, Thailand; 2 Center for Genomics and Bioinformatics Research, Faculty of Science, Prince of Songkla University, Hat Yai, Songkhla, Thailand; 3 Medical Technology Service Center, Faculty of Medical Technology, Prince of Songkla University, Hat Yai, Songkhla, Thailand; 4 Faculty of Medical Technology, Prince of Songkla University, Hat Yai, Songkhla, Thailand; Institute of medical research and medicinal plant studies, CAMEROON

## Abstract

Loss of β-cell mass and function is a fundamental feature of pathogenesis for type 1 and type 2 diabetes. Increasing evidence indicates that apoptosis is one of the main mechanisms of β-cell death in both types. Ethanolic extracts of *Pluchea indica* leaf (PILE) have been reported to possess blood glucose lowering actions *in vivo*. Nevertheless, further study is required to determine the underlying mechanisms. In this report, we have investigated the preventive effects of PILE on multiple low doses of streptozotocin (MLDS)-induced β-cell apoptosis. Mice were pre-treated with PILE at 50 mg/kg (PILE 50) or 100 mg/kg (PILE 100) for 2 weeks before streptozotocin (STZ) stimulation, and the treatment continued for 4 or 8 weeks. Results revealed that PILE 100 mice exhibited improved blood biochemistry, maintained a higher body weight, had decreased hyperglycemia, and restored islet architectures compared to non-treated STZ mice. Significantly, PILE 100 decreased levels of inflammatory response markers interferon-γ (IFN-γ), tumor necrosis factor-α (TNF-α), and interlukin1-β (IL-1β), concomitant with the inhibition of caspase-3, caspase-8, capsepase-9, phosphorylation of signal transducer and activator of transcription 1 (pSTAT1), nuclear factor-κBp65 (NF-κBp65), and inducible nitric oxide synthase (iNOS). Additionally, survival and proliferative ability of β-cells was mediated by up-regulated Bcl-2 and Ki67, respectively. These results provide strong evidence that pretreatment with PILE 100 effectively attenuated STZ-induced diabetes-related symptoms and these effects could be associated with the inhibition of cytokine-induced β-cell apoptosis.

## Introduction

Diabetes mellitus (DM) is a complex metabolic disorder characterized by the destruction of the insulin-secreting β-cells of the islets of Langerhans (T1DM) [1] or by insulin resistance whereby the insulin target organs are unresponsive to insulin action, which subsequently causes secondary β-cell damage due to prolonged exposure to high glucose levels (T2DM) [2]. Thus, despite of their etiopathogenetic differences, loss of β-cell number and function underlies much of the pathology of both T1DM and T2DM [1, 3]. Accumulating data suggests that both categories of diabetes share intra-islet expression of inflammatory mediators, particularly the cytokine interleukin (IL)-1β, activating a final common pathway of β-cell apoptosis through the nuclear factor (NF)-κB and Fas pathways, resulting in progressive β-cell loss in diabetes [4]. Hence, increasing β-cell mass by protecting it from cytokine-induced apoptosis is an effective method for protecting against and treating DM. This has been achieved with some success by pretreatment with various chemical compounds. For instance, inhibition of IL-1 expression by synthetic IL-1 receptor antagonist has been reported to protect islets against cytokine insult *in vitro* [5]. However, the synthetic chemicals have been tested by many groups with a limited degree of success due to the induction and overexpression of scavenging reactive oxygen species (ROS) enzymes in islets [5]. Moreover, it has been reported that these chemical compounds cause adverse side effects and cytotoxicity [6]. Therefore, the development of alternate, safe, and effective antidiabetic substances from natural sources and their systematic studies are as important as the development of new synthetic drugs.

*Pluchea indica* (Linn) Less. (*P*. *indica*), classified in the family Compositae (Asteraceae), is a plant that can be found in saline coastal areas around southeast Asia [7], including Thailand. The anti-inflammatory properties of *P*. *indica* root extracts have been reported since 1991 [7], and different parts (i.e., leaves, roots, and stems) of *P*. *Indica* have been used as major therapeutic agents to relieve ulcers and soothe sores [8]. Accumulating evidence further confirms that beneficial effects *P*. *Indica* leaf extracts range from antioxidant [9–11], anti-inflammatory [12, 13], and anti-cancer [10]. In Thailand, the fresh leaves of *P*. *Indica* are used as the main ingredient in many local dishes. Currently, dried leaves and leaf extracts have been commercially available as herbal tea due to its known blood glucose lowering properties [14, 15]. Although increasing scientific data has highlighted the antioxidant and antidiabetic effects of *P*. *Indica* leaf extracts, these reports are largely based on enzymatic activity evidence, such as α-glucosidase, α-amylase, and 2,2-diphenyl-1-picryl-hydrazyl-hydrate (DPHH) assays [11, 16, 17]. Therefore, the *in vivo* studies elucidating the mechanism of ethanolic *P*. *Indica* leaf extract (PILE) are of particular importance. Such studies are not only to validate the efficacy and safety of PILE consumption, but to increase traditional natural product use, thereby promoting general well-being for people who cannot easily access modern health care.

In this report, we tested whether PILE pretreatment could counteract with STZ-induced inflammatory responses and β-cell apoptosis. The multiple low doses of streptozotocin (MLDS) approach was used to induced β-cell apoptosis by triggering the appearance of T-cells, lymphocytic infiltration (insulitis), insulin deficiency, and finally hyperglycemia [18, 19]. The response under MLDS condition; therefore, more closely resembled T1DM in pathogenesis and morphological changes than the single high dose of STZ protocol [19]. In addition to blood biochemical analysis and histopathological examinations, proinflammatory cytokines (IFN-γ, TNF-α, and IL-1β), the intrinsic apoptotic pathway (caspase-9, and Bcl-2), and the extrinsic apoptotic pathway (caspase-3, caspase-8, pSTAT1, NF-κBp65, and iNOS) were detected to validate the protective effect of PILE and to clarify the mechanism of action in comparison with untreated STZ animals. Moreover, the beneficial effects of PILE were explored by detecting proliferation with a Ki67 marker.

## Materials and methods

### Collection and preparation of plant extract

Fresh leaves of *P*. *indica* were collected from Songkhla, Thailand. (7°6’26” N and 100°33’7E) and deposited at the Herbarium, Department of Biology, Faculty of Science, Prince of Songkla University (PSU Herbarium). The plant was verified and authenticated by a taxonomist and curator, Associate Professor Dr. Kitichate Sridith, and given a voucher specimen number to J.Nopparat-A.Nualla-ong 1 (PSU).

The plant material was dried in a hot air oven at 50°C overnight. After that, 10 g of dried leaves was extracted in an incubator shaker (ZWYR-200D, LABWIT, China) at 37°C for 3 days with 95% ethanol. The plant extract was concentrated and dried under reduced pressure using a rotary vacuum evaporator and filtered with 0.45 μm filters. *P*. *indica* leaf ethanolic extracts (PILE) were then stored at 4°C until future use. Different desired concentrations of PILE were prepared by dissolving with 5% (v/v) Tween 80 [11] before use in this study.

The chemical compositions of PILE were preliminarily screened using liquid chromatography-mass spectrometry (LC-MS) (Agilent Technologies). LC analysis and interpretation of mass-spectrum by cross-reference with the database of the National Institute Standard and Technology (NIST) were performed by the Scientific Equipment Center of Prince of Songkla University.

### Animal study

This study was conducted on 80 male BALB/C mice (5–6 weeks old), weighing approximately 20–22 g at the beginning of experiment. The animals were purchased from Nomura Siam International Co., Ltd., based in Bangkok, Thailand and were maintained under standard conditions of temperature (23 ± 2°C) and humidity (50 ± 10%) with an alternating 12-hour light/dark cycles in the animal facility of Prince of Songkla University. The experimental protocols described in this study was approved and guided by the Animal Ethics Committee of Prince of Songkla University. All animals were acclimatized under laboratory conditions for one week prior to experiments.

### Experimental design

After a 1-week adaptation period, mice were randomly assigned to 4 treatment groups as follows: (1) control group: non-STZ, received diluent (5% (v/v) Tween 80); (2) STZ group: STZ injection, received diluent; (3) PILE 50 +STZ group: STZ injection, fed with a dietary supplement of 50 mg/kg PILE; and (4) PILE 100 +STZ group: STZ injection, fed with a dietary supplement of 100 mg/kg PILE. After 2 weeks of pretreatment with the corresponding diet; diabetes was induced intraperitoneally for 5 consecutive days with either 50 mg/kg of STZ [20] (freshly prepared by dissolving in 0.1 M citrate buffer, pH 4.5) or citrate buffer alone (control animals). Week 0 was defined as the first day of STZ injection. Three days after the last STZ injection, fasting blood glucose level from the tail vein was examined using a blood glucose meter (Accu-Check Active and test strips, Roche diagnostic, Mannheim, Germany). Mice were considered diabetic when blood glucose levels were above 200 mg/dL [21, 22]. Mice continued to be fed with diluent, PILE 50 or PILE 100 mg/kg in diluent by gavage once daily for 4 weeks or 8 weeks. Body weights and fasting blood glucose levels were checked every week. Food and water were measured before administration daily. After the 4- and 8- week time points, mice were euthanized with overdose of thiopental (70 mg/kg b.wt.) to collect blood and remove the pancreas for further experiments.

### Blood biochemical assessments

Assessment of kidney and liver function markers was done by collecting blood samples (1 mL) from overnight fasting mice with a small cut at the tail tip and then allowing the blood to clot at room temperature (RT) for 30 min. Serum was collected by centrifugation at 2,000 xg at 4°C for 30 min and kept at -80°C until used. Serum triglycerides (TG), total cholesterol (TC), blood urea creatinine (BUN), creatinine, and albumin were measured using assay kits, following manufacturer’s instructions (Cell Biolabs, Inc., San Diego, CA, USA). In addition, the activities of serum aspartate aminotransferase (AST), alanine aminotransferase (ALT), and alkaline phosphatase (ALP) were estimated using standard methods on Cobas MIRA automated analyzer (Roche Diagnostics).

### Histological examination and insulitis determination

Immediately after removal, the pancreatic tissues were washed with ice-cold phosphate-buffered saline (PBS). The collected pancreas tissues were divided into 2 parts, either fixed in 10% buffered formalin for histological examination or processed for Western blot analysis. For histological analysis, tissue samples were processed for dehydration, clearing, and embedding in paraffin. Briefly, paraffin sections (5 μm thick) were put onto glass slides, deparaffinized in xylene, hydrated through graded ethanol to distilled water, and finally stained with hematoxylin and eosin (H&E). Morphometric evaluation of the pancreas (n = 6 sections/animal with 15 μm interval between each section form 6 animals/group) was determined quantitatively by light microscopy (Olympus D73 equipped with CellSens software) in a blinded fashion in regard to (a) insulitis score, determined according to a previous study by Citro et al. (2015) [23] using a scale of 0–4: score 0 referred to no insulitis, score 1 referred to peri-islet insulitis (sign of pathological infiltration changes restricted to the outside of islets), score 2 referred to intermediate insulitis (less than 25% of the islets showed sign of pathological infiltration), score 3 referred to intra-islet insulitis (between 25% and 75% of the islets showed sign of pathological infiltration changes), and score 4 corresponded to complete islet insulitis (more than 75% of the islets showed sign of pathological infiltration changes); (b) the relative value of the islet size, calculated by dividing the sum total islet area by the pancreas sectional area multiplied by 100; and (c) the number of islets per pancreas section determined by dividing the sum total number of islets by the pancreas sectional area multiplied by 100. The relative pancreatic weights were assessed by dividing the pancreatic wet weights by total body weight multiplied by 100 (n = 10/group).

### Immunofluorescent staining

For fluorescent microscopy imaging of insulin, cleaved-caspase 3, and Ki67, paraffin-embedded tissue sections were deparaffinized in xylene, hydrated through graded ethanol to distilled water, and permeabilized in PBS with 0.1% Triton X-100 (PBST) for 10 min. Blocking was performed using 5% bovine albumin serum (BSA) in PBST for 1 h at RT, followed by incubation with guinea pig polyclonal anti-insulin antibody (Abcam, Cambridge, UK) together with rabbit anti-cleaved caspase-3 (Cell Signaling Technology) or rabbit anti-Ki67 (Abcam) diluted 1:100 in 1% BSA in PBST at 4°C overnight. Three consecutive washes with PBST for 5 min each were followed by sequential incubation with either Alexa Fluor 488 goat anti-guinea pig or Alexa Fluor 594 goat anti-rabbit secondary antibodies (Invitrogen) diluted 1:300 in 1% BSA in PBST at RT for 1 h. The slides were washed 3 times with PBST and mounted using a prolonged diamond anti-fade mounting medium containing 4',6-diamidino-2-phenylindole (DAPI) (Invitrogen). Images were captured under a fluorescence microscope (Olympus D73 equipped with CellSens software). Positive staining for insulin in pancreatic sections was quantified at 630-fold magnification and expressed as the percentage of insulin-positive cells divided by islet area multiplied by 100. In addition, quantification of the percentage of islets containing apoptotic β-cells was performed by the manually counted number of cleaved-caspase 3 positive staining cells divided by the total number of DAPI stained cells multiplied by 100. Lastly, the percentage of cell proliferation was determined by the number of coexpressing insulin and Ki67 cells divided by the insulin fluorescent intensity within each islet core. National Institutes of Health (NIH) ImageJ software was employed to measure fluorescent intensity. At least 5 different islets per pancreas section and 3 to 4 mice per condition were counted.

### Western blot analysis

Antibodies were obtained from the following sources: TNF-α, IL-β, caspase-3, caspase 8, caspase 9, NF-κBp65, pSTAT1, and iNOS (Cell Signaling Technology); IFN-γ and β-actin (Abcam). The pancreatic lysates were prepared on ice-cold radioimmunoprecipitation assay (RIPA) buffer (Sigma-Aldrich, St. Louis, MO, USA) supplemented with 1x protease inhibitor cocktails (Merk-Millipore, Bedford, MA, USA). Tissue lysates were then cleared by centrifugation at 14,000 xg for 30 min at 4°C. Total protein concentration was determined by BCA protein assay kit (Pierce biotechnology, Rockford, IL, USA). Approximately 60 μg proteins from each sample were separated on 10–12% SDS/PAGE and the proteins were transferred to polyvinylidene difluoride (PVDF) membranes (Merk-Millipore). After blocked in blocking buffer (5% non-fat dry milk in TBS containing 0.05% Tween 20; TBST) for 1.5 h at RT, membranes were then probed with the primary antibody overnight in blocking buffer (1: 1,000). After washing 3 times, the membranes were incubated for 1 h with horseradish peroxidase (HRP)-linked secondary antibodies (Sigma-Aldrich) in blocking buffer (1: 5,000). The level of β-actin was estimated in samples to check for equal loading of samples followed by washing 3 times with TBST. Finally, antigen-antibody complexes were observed using Luminata Crescendo Western HRP substrate (Merk-Millipore) according to the manufacturer's instructions. The density of the immunoreactive bands was analyzed using NIH ImageJ software. Data were expressed as fold change compared to normal control group and then statistical analysis was conducted.

### Statistical analysis

The results are presented as means ± standard error of the mean (SEM) unless noted otherwise. Statistical analyses were performed using one-way or two-way analysis of variance (ANOVA) followed by Tukey’s multiple comparisons with the GraphPad Prism 7.0 (GraphPad Software, San Diego, CA). Statistical significance was set at the 0.05 level.

## Results

### LC-MS analysis of phytochemical constituents of PILE

**Fig 1:**
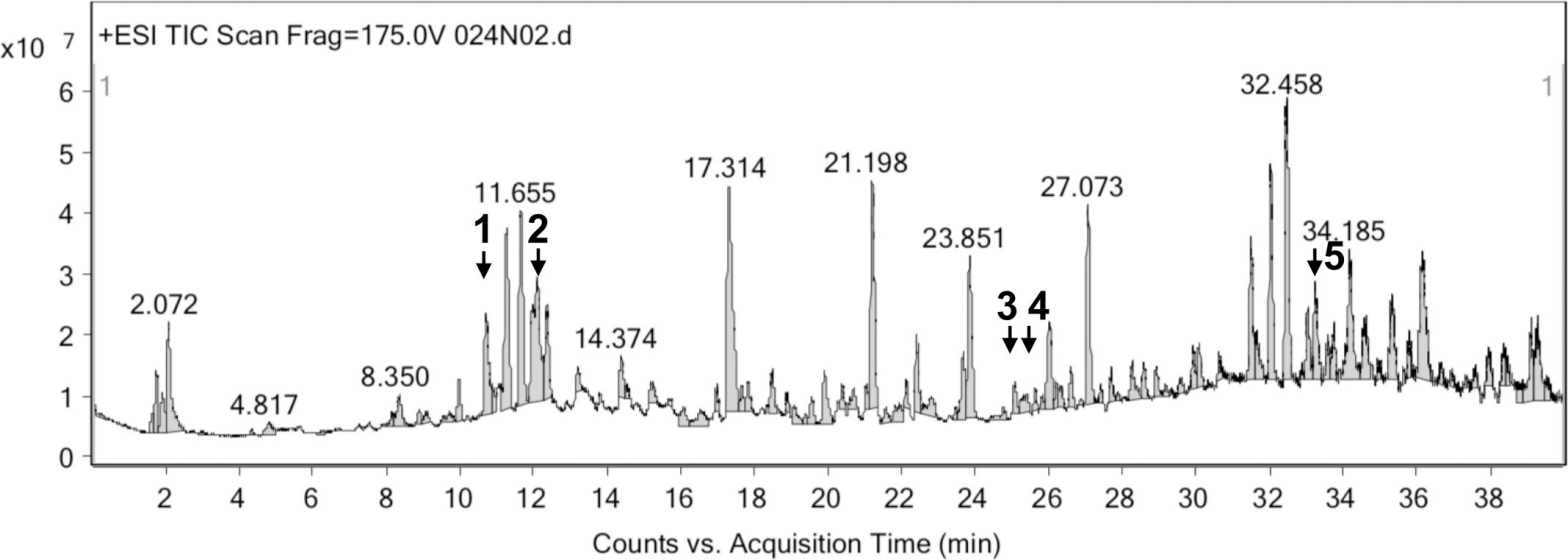
LC-MS chromatogram of *Pluchea indica* crude leaf ethanol extracts (PILE).

The significant compounds of PILE analyzed by LC-MS are summarized in Table 1. The LC-MS chromatogram is shown in Fig 1. Major flavomoids in PILE are 8-hydroxyluteolin 8-glucoside and 6-hydroxykaempferol 7-glucoside, trans-trismethoxy resveratrol-d4, and quercetin. Bioactive compounds in phelolic acids including 3,4-dicaffeoyl-1,5-quinolactone, apigenin 7-(2'',3''diacetylglucoside), and campesteryl ferulate were also detected in PILE. The chemical structures of these compounds characterized by LC-MS method are shown in S1 File. The preliminary phytochemical screening and the list of identified compounds of PILE analyzed by GC-MS are depicted in Figure A in S2 File and Table A in S2 File, respectively.

**Table 1:** LC-MS detection of phytoconstituents isolated from *Pluchea indica* crude leaf ethanol extracts (PILE).

Peak number	Retention time (min)	Identified compound name		Component area	%
		Flavonoids	Phenolic acids		
1	10.935	Quercetin	-	16,965,719.32	0.26
2	11.192	6-Hydroxykaempferol 7-glucoside	-	248,616,370.99	3.85
	11.192	8-Hydroxylueolin 8-glucoside			
3	12.238	-	3,4-Dicaffeoyl-1,5-quinolactone	8,212,657.17	0.12
4	12.479	-	Apigenin 7-(2'',3''diacetylglucoside)	114,322,902.05	1.77
5	25.369	Trans-trismethoxy resveratrol-d4	-	38,489,156.85	0.59
6	34.606	-	Campesteryl ferulate	45,402,529.96	0.70

### Effect of PILE pretreatment on blood biochemical analysis

First and foremost, we determined the blood biochemical analysis of some markers related to kidney and liver functions in order to validate whether administration of PILE could produce toxicity to experimental animals. No significant variance in serum TG, TC, BUN, creatinine, albumin, AST, ALT and ALP was observed among all experimental groups before MLDS stimulation (Table 2), indicating the safety of PILE administration. However, 4 weeks after MLDS treatment, as expected, mice showed significant increases in most parameters in comparison to control group, with the exception of TC and albumin, which showed slight changes in all animals tested here. Meanwhile, pretreatment with PILE at a dose of 50 mg/kg had little effect on preventing STZ toxicity. By contrast, the higher dose (PILE 100) improved the above parameters. AST, ALT, and ALP levels were reduced significantly in PILE 100 mouse serum compared to that of untreated STZ animals, suggesting its ameliorating effect on hepatocellular functions. Although, we failed to detect significant changes statistically, BUN and creatinine in PILE treatment tend to be lower than that of STZ animals.

**Table 2:** Effects of pretreatment with PILE for 10 weeks on blood biochemistry parameters of STZ-induced diabetic mice.

Parameters	Weeks	Control	STZ	PILE		*P1*	*P2*	*P3*
				50 mg/kg	100 mg/kg			
TC	-2	111.0 ± 11.2	103.6 ± 4.5	103.3 ± 16.0	108.6 ± 19.2	0.914	>0.999	0.970
(mg/dL)	0	107.0 ± 5.5	106.6 ± 7.0	117.0 ± 14.1	101.0 ± 6.5	0.9963	0.2035	0.6408
	4	106.0 ± 9.0	153.0 ± 10.2	123.0 ± 16.3	129.3 ± 5.6	0.0036	0.0418	0.1113
	8	113.3 ± 14.5	161.6 ±12.1	125.6 ± 10.9	133.6 ±21.1	0.0192	0.0764	0.1877
TG	-2	105.3 ± 10.6	127.6 ± 15.0	123.3 ± 4.6	108.0 ± 14.0	0.8952	0.7015	0.9713
(mg/dL)	0	115.0 ± 8.6	116.3 ± 24.2	115.3 ± 12.0	124.0 ± 12.1	0.9998	>0.9999	0.9981
	4	116.3 ± 8.3	155.3 ± 34.4	126.6 ± 12.0	130.0 ± 17.3	0.1732	0.3819	0.4777
	8	111.3 ± 8.3	178.0 ± 26.6	130.0 ± 11.5	128.6 ± 14.5	0.0054	0.0326	0.0285
BUN	-2	16.0 ± 1.7	18.67 ± 1.1	17.6 ± 3.5	16.3 ± 1.5	0.4808	0.9406	0.5815
(mg/dL)	0	22.0 ± 8.1	21.3 ± 7.5	21.0 ± 2.6	19.6 ± 7.2	0.9993	>0.9999	0.9898
	4	20.3 ± 5.5	43.6 ± 5.1	28.3 ± 4.1	26.6 ± 11.0	0.0148	0.1034	0.0685
	8	17.3 ± 3.0	41.0 ± 6.2	27.0 ±11.0	25.6 ± 14.5	0.042	0.2752	0.2153
Creatinine	-2	0.4 ± 0.1	0.41 ± 0.1	0.5 ± 0.1	0.4 ±0.1	0.9979	0.9906	0.9931
(mg/dL)	0	0.7 ± 0.2	0.6 ± 0.1	0.6 ± 0.2	0.6 ± 0.1	0.9633	0.9633	>0.9999
	4	0.5 ± 0.2	1.6 ± 0.1	0.8 ± 0.2	0.7 ± 0.2	0.002	0.0032	0.002
	8	0.6 ± 0.1	1.8 ± 0.5	1.07 ± 0.5	1.0 ± 0.4	0.0152	0.1054	0.0895
Albumin	-2	2.5 ± 0.1	2.3 ± 0.6	2.4 ± 0.1	2.4 ±0.1	0.9791	0.9791	0.9953
(g/dL)	0	2.3 ± 0.3	2.43 ± 0.2	2.6 ± 0.2	2.5 ± 0.3	0.9755	0.7787	0.9924
	4	2.2 ± 0.3	3 ± 0.2	2.3 ± 0.6	2.4 ± 0.4	0.1748	0.2571	0.3688
	8	1.8 ± 0.2	2.4 ± 0.3	2.07 ± 0.5	1.8 ± 0.5	0.0236	0.0754	0.0314
AST	-2	109.6 ± 9.7	112.7 ± 10.7	114.0 ± 12.5	111.6 ± 10.7	0.9872	0.9988	0.9995
(IU/L)	0	112.0 ± 9.8	109.0 ± 12.4	113.3 ± 13.8	109.0 ± 12.4	0.9903	0.972	0.972
	4	108.7 ± 9.4	236.6 ± 34.5	191.6 ± 48.9	177.6 ± 48.5	0.0159	0.5228	0.3154
	8	110.6 ± 13.6	295.0 ± 54.8	187.0 ± 37.8	184.0 ± 42.5	0.0022	0.043	0.0187
ALT	-2	42.6 ± 9.0	33.3 ± 5.5	36 ±7.2	32.0 ± 8.0	0.4723	0.9711	0.9961
(IU/L)	0	51.0 ± 7.5	50.3 ± 3.7	49.6 ± 8.7	23.0 ± 3.6	0.9981	>0.9999	>0.9999
	4	61.6 ± 8.0	220.0 ± 11.1	168.6 ± 54.0	170.0 ± 48.5	0.0034	0.3825	0.4027
	8	59.0 ± 10.4	244.0 ± 54.4	123.3 ± 43.1	121.0 ± 40.4	0.0023	0.0269	0.0244
ALP	-2	82.6 ± 6.1	75.6 ± 7.3	82 ±13.1	82.0 ± 5.2	0.7517	0.8012	0.8012
(IU/L)	0	97.3 ± 4.0	100.3 ± 15.0	94.3 ±13.0	108.0 ± 5.2	0.9842	0.8943	0.8082
	4	95.0 ± 7.0	149.3 ± 20.3	118.0 ± 12.1	112.3 ± 4.7	0.0031	0.0593	0.0272
	8	100.5 ± 5.5	164.3 ± 17.6	115.3 ± 7.0	114.6 ± 10.2	0.0002	0.0014	0.0013

TC; Total cholesterol; TG, Triglyceride; BUN, Blood Urea Nitrogen, AST, Aspartate Aminotransferase; ALT, Alanine Aminotransferase, ALP, Alkaline Phosphatase. Control: untreated group; STZ: diabetic control group; PILE 50 + STZ: 50 mg/kg/day of PILE pretreated group with concomitant MLDS stimulation: PILE 100 + STZ: 100 mg/kg/day of PILE treated group with concomitant MLDS stimulation. Values represent the means ± S.D. (n = 3 per group). The *P1* values are derived from the STZ group compared with control, while *P2* and *P3* values indicate the differences between the STZ vs. PILE 50 + STZ and PILE 100 + STZ, respectively. A *P* value of < 0.05 is considered statistically significant.

### PILE pretreatment reduces STZ-induced physiological and physical characteristics associated with diabetes

**Fig 2:**
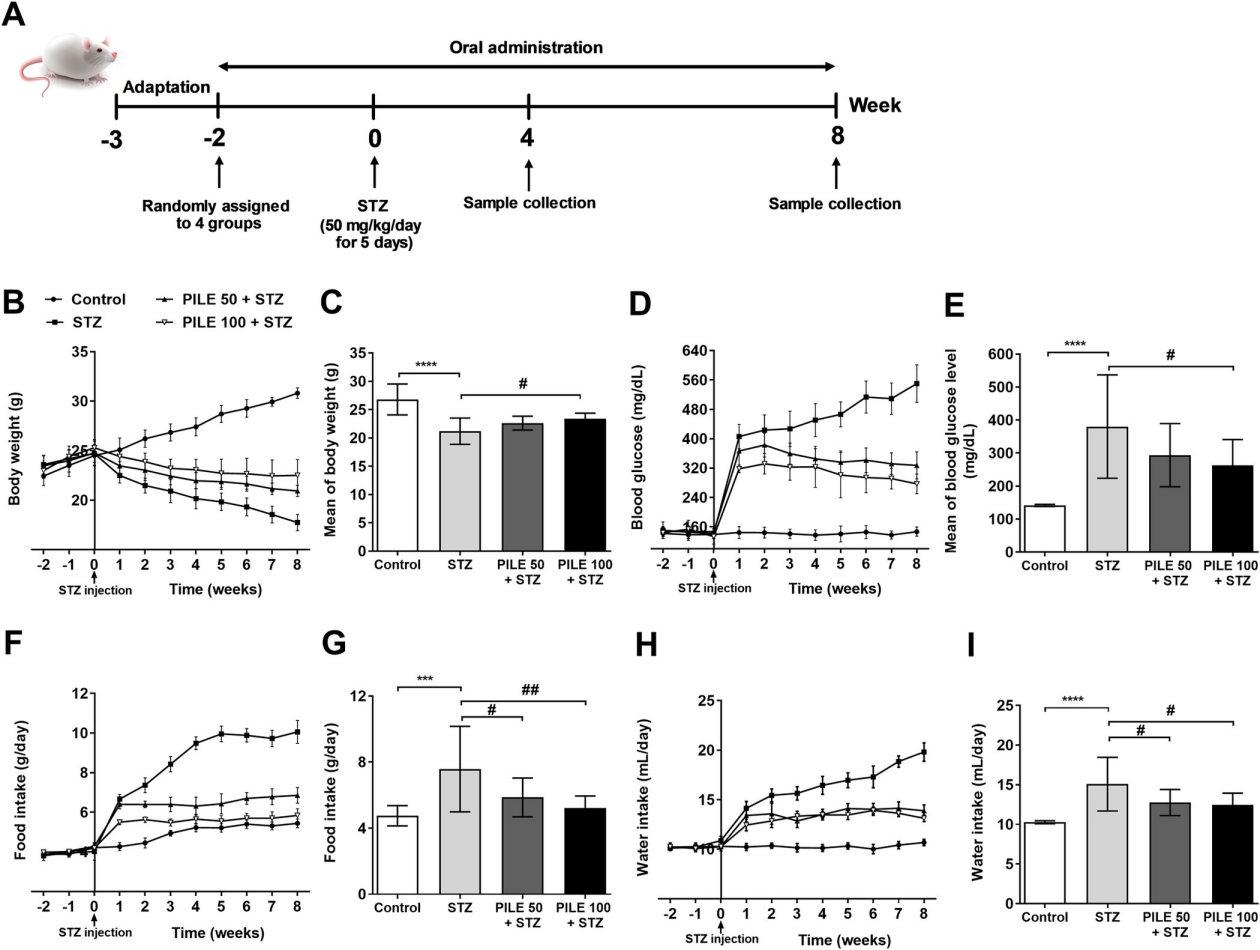
PILE pretreatment ameliorates physiological and feeding behavior changes in STZ mice. Time line of the study (A), changes of (B) and (C) body weights, (D) and (E) fasting blood glucose levels, (F) and (G) food intake, and (H) and (I) water intake of the experimental mice throughout the entire study (10 weeks). PILE and diluent were administered daily for two weeks before the induction of diabetes by MLDS (50 mg/kg. b.wt.) for 5 consecutive days, and the treatment was continued for 8 weeks. All parameters were significantly lower in PILE 100 + STZ pretreatment groups compared with the values of STZ untreated groups, indicating the efficacy of PILE pretreatment against STZ cytotoxicity. Control: untreated group; STZ: diabetic control group; PILE 50 + STZ: 50 mg/kg/day of PILE pretreated group with concomitant MLDS stimulation: PILE 100 + STZ: 100 mg/kg/day of PILE treated group with concomitant MLDS stimulation. Values are the mean ± S.D. (n = 10 mice per group). Data were analyzed by one-way ANOVA followed by Tukey’s multiple comparison test. ****P < 0.0001, ***P < 0.001 vs. the corresponding control group; ^##^*P* < 0.01, ^#^*P* < 0.05 vs. the corresponding STZ group.

Prior to MLDS stimulation, the average of body weight, blood glucose, and feeding behavior values were equivalent among all groups (Fig 2). Injection of STZ for 5 consecutive days resulted in a dramatic reduction of the mean body weight (21.18 ± 2.32), caused a sharp elevation of the mean blood glucose (380.31 ± 156.60 mg/dL) as well as induced progressive polyphagia (7.58 ± 2.58 g/day) and polydipsia (15.07 ± 3.38 ml/day) in untreated STZ animals. However, STZ cytotoxicity was significantly less pronounced in the PILE 100 pretreated mice when compared with the untreated animals. Notably, the blood glucose levels of PILE 100 mice moderately increased after MLDS administration (262.68 ± 78.34 mg/dL) and ranged from 276–331 mg/dL. Meanwhile, untreated STZ mice displayed a significantly higher range of blood glucose levels (406–550 mg/dL) and remained higher than 400 mg/dL throughout the experimental period. These results demonstrated that PILE at 100 mg/kg significantly attenuates MLDS-induced physiological and physical characteristics associated with diabetes.

### PILE pretreatment attenuates STZ-induced islet injuries and inflammatory responses in the pancreases of mice

**Fig 3:**
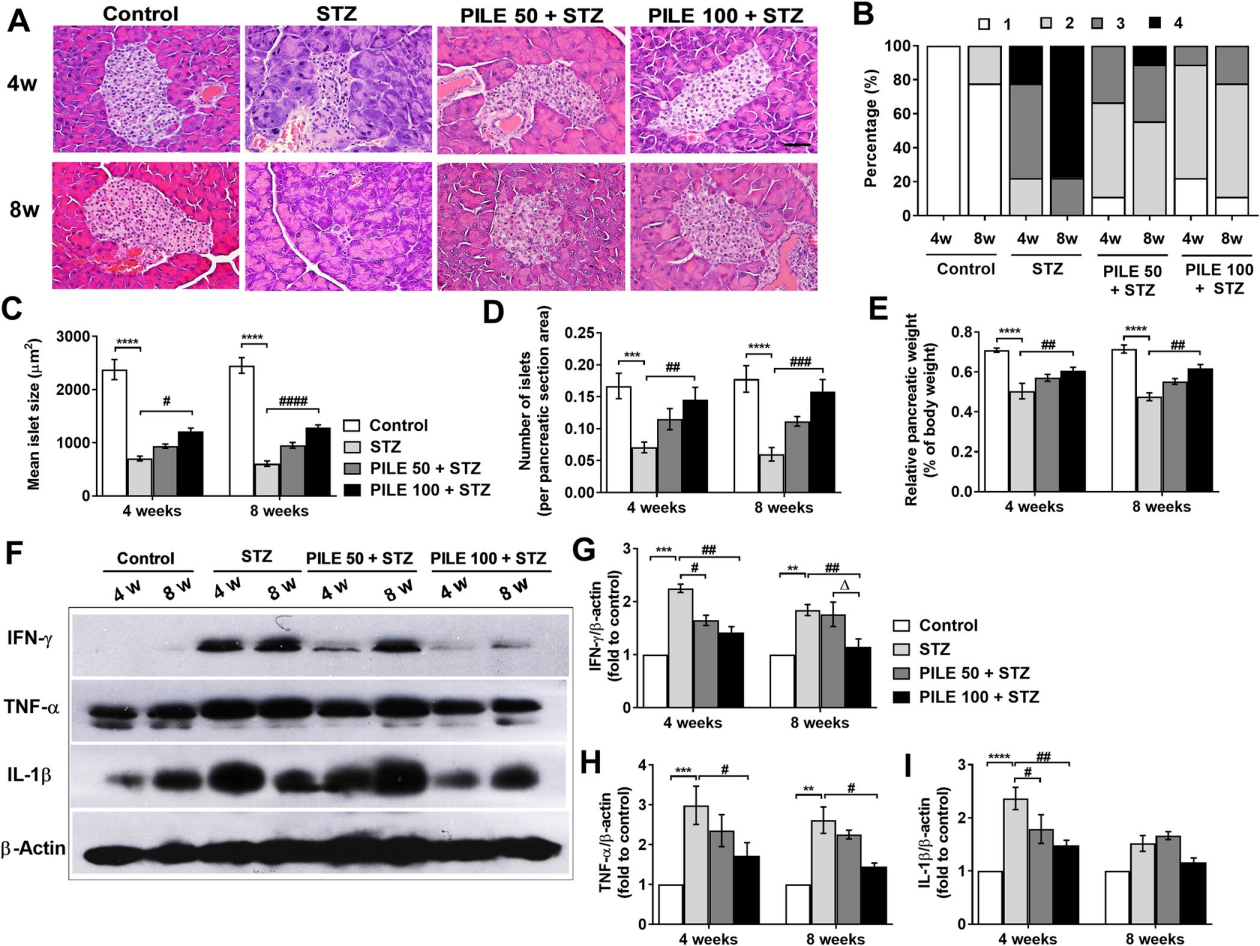
PILE pretreatment attenuates STZ-induced histopathological changes and cytokines profiles in the pancreases of mice. (A) Representative images of the pancreatic tissues from different groups were examined using H&E staining. All images were taken at 400 magnification. Scale bars, 20 μm. (B) Insulitis was reduced by PILE pretreatment in diabetic mice. The percentage of islets in each of the 4 categories was as follows: 0, no insulitis; 1, peri-islet insulitis; 2, intermediate insulitis; 3, intra-islet insulitis; and 4, severe insulitis. (C) Distribution of islet sizes among experimental groups. (D) The average number of islets per cross-sectional areas of all islets found in each group. (E) Percentage of pancreatic wet weights normalized to total body weight. (F) Representative images of Western blot analysis of pancreatic cytokines IFN-γ, TNF-α, and IL-1β. (G)—(I) Densitometry quantification of the IFN-γ, TNF-α, and IL-1β corrected by β-actin. Control: untreated group; STZ: diabetic control group; PILE 50 + STZ: 50 mg/kg/day of PILE pretreated group with concomitant MLDS stimulation: PILE 100 + STZ: 100 mg/kg/day of PILE treated group with concomitant MLDS stimulation. Data were obtained from 36 histological stained sections per 6 mice per group for H&E examination and n = 3 per group for Western blotting assay. Values represent means ± SEM. ****P < 0.0001, ***P < 0.001, **P < 0.01 vs. the corresponding control group; ^####^*P* < 0.0001, ^###^*P* < 0.001, ^##^*P* < 0.01, ^#^*P* < 0.05 vs. the corresponding STZ group; ^Δ^*P* < 0.05 vs. the corresponding PILE 50 group.

Consistent with the changes of body weight and blood glucose levels, untreated STZ mice displayed severe pancreatic islets morphology. The islets appeared shrunken, with ill-defined borders and higher insulitis scores observed in experimental diabetic mice at the 4- and 8-week time points (Fig 3A and 3B). Moreover, the islet size and number were dramatically decreased in STZ mice versus those in the control group (Fig 3C and 3D). Even though the prevention of insulitis in PILE-treated mice was not complete, PILE pretreatment potentially preserved the above pathological changes (Fig 3A–3D). Moreover, as shown in Fig 3E, compared with the control group, exposure to STZ had a significant impact on relative pancreatic weights, suggesting pancreatic atrophy, but pretreatment with PILE 100 significantly relieved this STZ toxicity.

we further investigated the changes in cytokine-mediated inflammatory responses, which are the underlying mechanisms involved in the severity of STZ-induced islet dysfunction. Our results show the significant increase of IFN-γ, TNF-α, and IL-1β expressions in pancreatic lysates in STZ-induced diabetic mice, compared to those from the control group (Fig 3F–3H). Nevertheless, PILE pretreatment effectively counteracted each inflammatory cytokine in a dose- and time-dependent manner. We found that IFN-γ played an essential role in our mouse model, and PILE 50 and PILE 100 significantly reduced IFN-γ production in the pancreas at both time points when compared with untreated STZ mice (Fig 3F and 3G). Meanwhile the suppression of TNF-α was statistically significant with a dose of PILE 100, but not PILE 50. IL-1β production was only significantly elevated after exposure of the mice to STZ for 4 weeks and became weaker and remained unchanged in all experimental groups observed at the 8-week time point (Fig 3F and 3I). Our observations suggest that administration of PILE may provide a certain degree of β-cell protection at least through the suppression of proinflammatory cytokine production in the pancreas of STZ-induced diabetic model mice.

### PILE pretreatment helps to prevent STZ-induced β-cell apoptosis in mice

**Fig 4:**
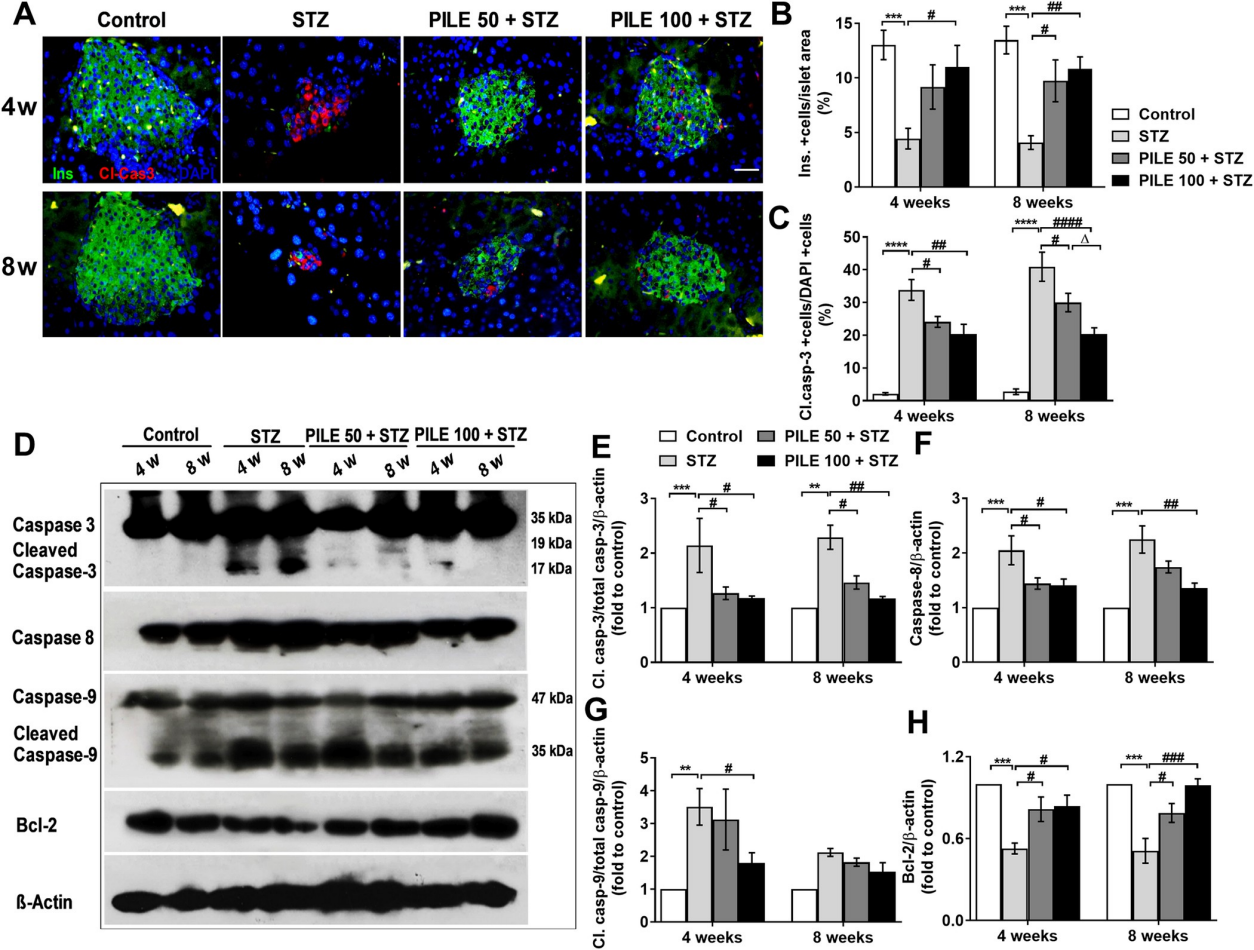
PILE pretreatment preserved β-cells by inhibiting the apoptotic pathway in STZ mice. (A) Representative immunofluorescent images of islets by cleaved caspase-3 (red), β-cell marker insulin (green) and DAPI (blue) staining in sections of formalin-fixed paraffin-embedded among experimental groups at the 4- and 8-week time points. (B) Quantification of the percentage of islets containing insulin positive cells per islet area. All images were taken at 630 magnification. Scale bars, 20 μm. (C) Quantification of the percentage of active caspase 3-positive cells/total insulin fluorescent intensity area. Data represent the mean ± SEM from measurements of approximately 15–20 independent islets per group (5 islets per mouse pancreas were randomly chosen from 3–4 animals per group). (D) Representative images of pancreatic protein expressions of total caspase-3 and cleaved caspase-3, caspase 8, total caspase-9 and cleaved caspase-9, and Bcl-2 assessed by Western blotting. (E)—(H) Densitometry quantification of the cleaved-caspase3/total caspase 3, caspase 8, cleaved-caspase9/total caspase 9, and Bcl-2 corrected by β-actin. Control: untreated group; STZ: diabetic control group; PILE 50 + STZ: 50 mg/kg/day of PILE pretreated group with concomitant MLDS stimulation: PILE 100 + STZ: 100 mg/kg/day of PILE treated group with concomitant MLDS stimulation. Values represent means ± SEM (n = 3 per group). ****P < 0.0001, ***P < 0.001, **P < 0.01 vs. the corresponding control group; ^####^*P* < 0.0001, ^###^*P* < 0.001, ^##^*P* < 0.01, ^#^*P* < 0.05 vs. the corresponding STZ group; ^Δ^*P* < 0.05 vs. the corresponding PILE 50 group.

It is well-documented that STZ selectively targets β-cells, and with the MLDS approach, the presence of cytokines could further induce cell death through apoptosis. By immunohistochemistry, untreated STZ mice displayed a major reduction in insulin-positive staining cells (Fig 4A and 4B) concomitant with a significant increase in apoptotic β-cells by active caspase-3 staining at both the 4- and 8-week time points compared to that of control mice (Fig 4A and 4C). In contrast, both PILE 50 and PILE 100 significantly reduced apoptosis of β-cells (Fig 4A–4C).

We also performed Western blot analysis in order to define target molecules of PILE treatment in the apoptotic pathway. Compared with the control group, mice in the STZ group displayed a significant elevation of cleaved-caspase 3, caspase 8, and cleaved-caspase-9 at the 4- and 8-week time points (Fig 4D–4G). However, both doses of PILE pretreatment showed notable suppression of cleaved-caspase 3, and its protective effect extended to 8 weeks. Meanwhile, PILE 50 had a less pronounced effect on caspase-8 in comparison with PILE 100, especially at the 8-week time point. Moreover, a significant decrease of cleaved-caspase 9 was detected in PILE 50- and PILE 100-treated mice at the 4-week time point, but a similar beneficial effect was not statistically different at the 8-week time point.

We took a closer examination by investigating the protein level of Bcl-2, an anti-apoptotic molecule (Fig 4D and 4H). The results showed that STZ stimulation significantly decreased the Bcl-2 production in the pancreas, whereas pretreatment with PILE 50 and PILE 100 remarkably preserved Bcl-2 production to nearly normal levels. These results demonstrate the important role of PILE pretreatment in preventing β-cell apoptosis *in vivo*.

### PILE pretreatment potentially induces cell proliferation in the pancreases of STZ-induced diabetic mice

**Fig 5:**
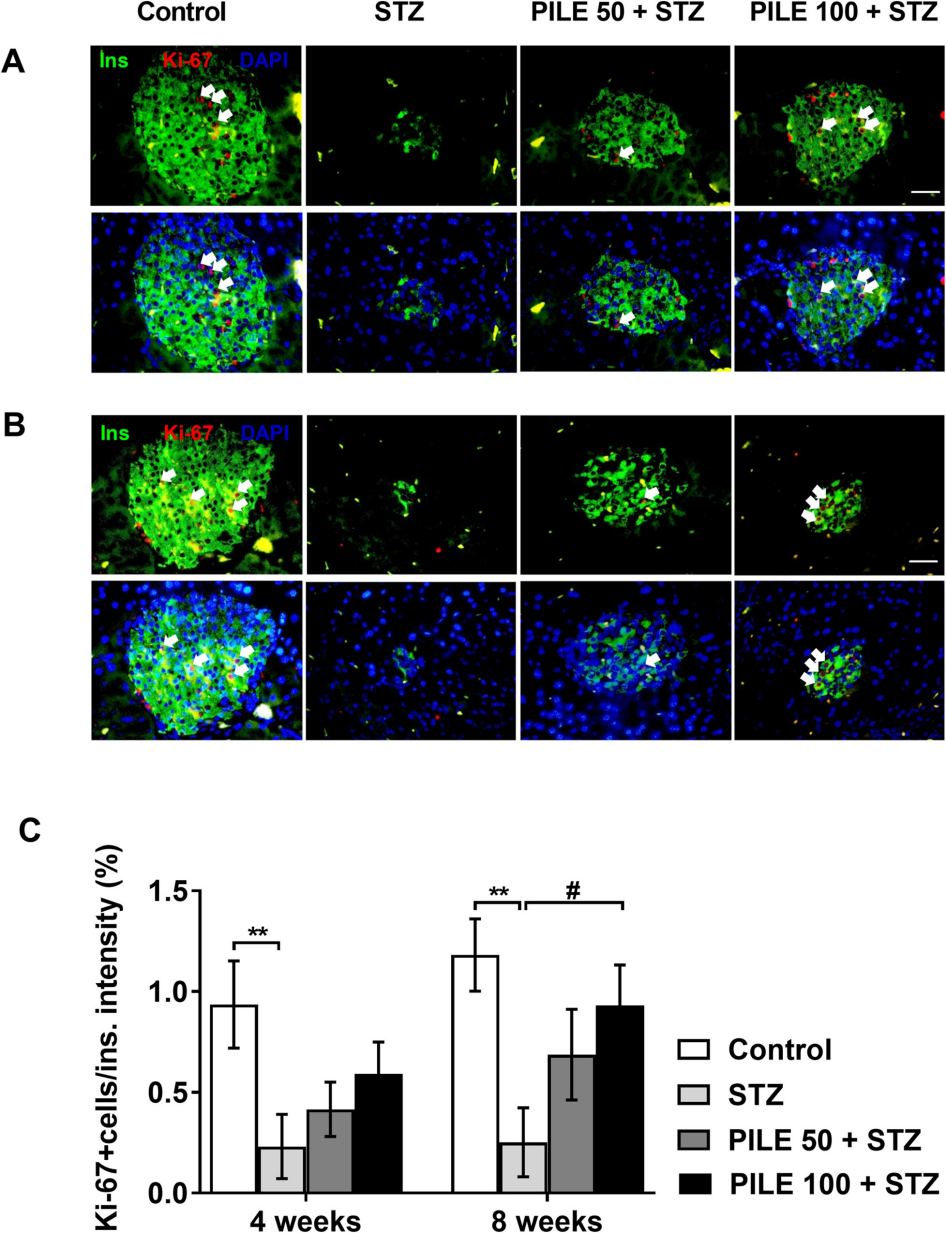
PILE pretreatment potentially induces β-cell proliferation in STZ mice. Representative immunohistochemistry images from each group for proliferation of β-cells in islets from the (A) 4-week time point to the (B) 8-week time point using immunostaining in sections of formalin-fixed paraffin-embedded with Ki67 antibody (red), β-cell marker insulin (green). Counterstain DAPI (blue) marks the nuclei. White arrows mark the proliferating β-cells, labeled by Ki67 and insulin. (C) Quantification of the percentage of Ki67 positive cells per insulin fluorescent intensity. Control: untreated group; STZ: diabetic control group; PILE 50 + STZ: 50 mg/kg/day of PILE pretreated group with concomitant MLDS stimulation: PILE 100 + STZ: 100 mg/kg/day of PILE treated group with concomitant MLDS stimulation. All data are mean ± SEM of n = 3 mice per group, 15–20 islets per group. **P < 0.01 vs. the corresponding control group; ^#^*P* < 0.05 vs. the corresponding STZ group.

To determine whether the blood glucose lowering effect of PILE pretreatment was solely the result of inhibition of β-cell death, or whether there was also an effect on β-cell proliferation, pancreatic tissues were stained for the proliferative marker Ki67. As depicted in Fig 5A and Fig 5B, colocalization of Ki67 and insulin-positive β-cells (white arrows) was significantly diminished in untreated STZ mice compared with that of control animals. Pretreatment with PILE 100, but not PILE 50, significantly improved β-cell proliferation at 8-week time point only.

### PILE pretreatment improves the execution molecules in cytokine-induced β-cell apoptosis in STZ-induced diabetic mice

**Fig 6:**
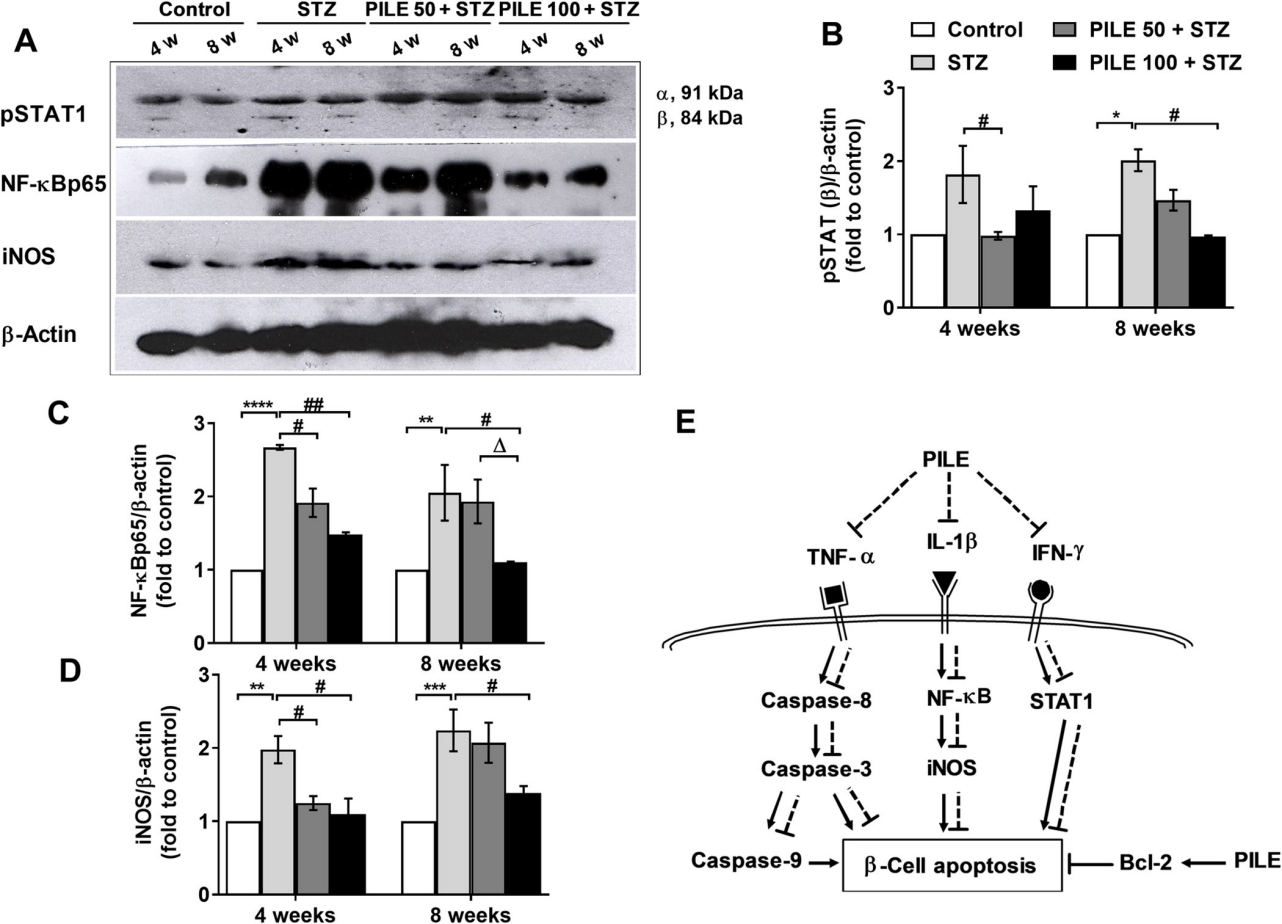
PILE pretreatment counteracts the cytokine-induced β-cell toxic singling pathway. (A) Representative images of pancreatic protein expressions of pSTAT1, NF-κBp65, and iNOS were measured by western blotting. (B)—(D) Densitometry quantification of pSTAT1, NF-κBp65, and iNOS normalized to β-actin. (E) A proposed potential inhibitory mechanism of MLDS-induced β-cell apoptosis by PILE. MLDS in mice induces infiltration of cytokines with selective destruction of β-cells and promotes cytokine-initiated apoptotic signaling. PILE could inhibit the expressions of IFN-γ, TNF-α, and IL-1β, leading to inhibition of the NF-κB pathway and caspase-3 activation, which subsequently protects the β-cells. In addition, PILE potentially increases Bcl-2 expression, which in turn promotes β-cell survival. Solid lines indicate the stimulatory actions by STZ or PILE. Dashed lines indicate the inhibitory actions by PILE. Control: untreated group; STZ: diabetic control group; PILE 50 + STZ: 50 mg/kg/day of PILE pretreated group with concomitant MLDS stimulation: PILE 100 + STZ: 100 mg/kg/day of PILE treated group with concomitant MLDS stimulation. Values represent means ± SEM (n = 3 per group). ****P < 0.0001, ***P < 0.001, **P < 0.01, *P < 0.05 vs. the corresponding control group; ^#^*P* < 0.05 vs. the corresponding STZ group; ^Δ^*P* < 0.05 vs. the corresponding PILE 50 group.

Increasing evidence suggests that β-cells respond to the cytokines IL-1β, TNF-α, and IFN-γ by modifying the expression of the apoptotic signaling cascade under the regulation of two master transcription factors, including STAT1 and NF-κBp65. We found that STZ significantly induced pSTAT-1 (β isoform) (Fig 6A and 6B) and NF-κB65 (Fig 6A and 6C) levels at the 4- and 8- week time points versus the control group, consistent with cytokine protein expression profiles (Fig 3F). Nevertheless, PILE 50 and PILE 100 pretreatments greatly inhibited pancreatic levels of pSTAT1 only at 4-week time point. Moreover, both doses of PILE pretreatment showed a remarkable inhibitory effect on NF-κB65 level throughout the experimental period (Fig 6C).

Since iNOS is an important downstream effector of activated NF-κBp65, we also examined the expression of iNOS among experimental groups to identify whether PILE would display a similar beneficial effect. Results showed that, compared with control animals, STZ mice exhibited a significant increase of iNOS, indicating its relation to NF-κBp65 activation (Fig 6D). Pretreatment with PILE 50 showed significantly lower iNOS production only at the 4-week time point, whereas PILE 100 showed a preventive effect on iNOS continuously until the 8-week time-point (Fig 6D).

## Discussion

In the present study, we present original evidence that pretreatment with PILE does modify the response of the pancreas to the cytotoxicity of MLDS stimulation in three ways. Firstly, PILE could reduce the infiltration of pro-inflammatory cytokines into pancreatic tissue. Consequently, there was less β-cell damage by the cytokines; therefore, some β-cells were permitted to survive. This phenomenon in such pre-treated animals is also reflected functionally by the maintaining a higher body weight, lesser degree of hyperglycemia, improved islet morphology, and β-cell numbers. Secondly, PILE pretreatment prevents the activation of both intrinsic (mitochondrial driven) and extrinsic (receptor-mediated) β-cell apoptotic pathways. Both of these underlying mechanisms could prevent β-cell death. Lastly, PILE pretreatment promotes β-cell survival through up-regulation of the anti-apoptotic protein Bcl-2 expression in conjunction with an increased Ki67 protein, a marker for cell proliferation.

*P*. *indica*’ s fresh leaves and leaf extracts have been used traditionally to relieve various diseases as they are documented to possess wide range of biological properties namely antioxidant [9,10,14], anti-inflammatory [8, 12], anti-ulcer [8], and anti-tuberculosis [24]. Nevertheless, the preventive effects of PILE on cytokine-induced β-cell death in diabetic animal models have not yet been investigated. In this experiment, PILE 100 mice compared to STZ mice over the time course significantly reduced the increase of the blood glucose level, suggesting its hypoglycemic effect. Comparable findings were reported by Kesari et al. (2005) [25] and Widyawati et al. (2015) [26]. In fact, it was note that *P*.*indica* extracts exhibited α-glucosidase inhibitory effect [11, 16]. By inhibiting this enzyme, the breakdown of starch into glucose can be delayed and could lead to the increase of glucose uptake of circulating glucose, thereby lower the blood glucose level [16]. Another plausible explanation of the results of the present work could be attributed to the phytochemicals of PILE. The major phytoconstituents detected in PILE by LC-MS include phenolic acids (3,4-dicaffeoyl-1,5-quinolactone, apigenin 7-(2'',3''diacetylglucoside), and campesteryl ferulate) and flavonoids (quercetin, 6-hydroxykaempferol 7-glucoside, 8-hydroxy- luteolin 8-glucoside, and trans-trismethoxy resveratrol-d4). These results are in accordance with other works reporting that this plant has high contents of phenolic acids and flavonoids, which are well-known for their antioxidant and anti-inflammatory activities [11, 27, 28]. On the basis of literature and our experimental results, we presumed that the presence of quercetin and trans-trismethoxy resveratrol-d4 (resveratrol derivative) could significantly attribute to the hypoglycemic activity of PILE. These bioactive compounds have been reported to exert their antidiabetic effects by a) acting as free radical scavengers [29]; b) improving liver carbohydrate metabolism [30]; and c) improving glucose uptake through mediators of the insulin-signaling pathways [31, 32]. Notably, quercetin has been reported to have higher antioxidant activity compared to other well-known antioxidant molecules namely ascorbly and trolox because of the number and positions of the free hydroxy groups in its structure [33]. Therefore, it is presumable that blood glucose-lowering activity of PILE presented in the current study is strongly related to the antioxidant activities of extract’s phytochemicals, specifically quercetin and resveratrol derivative.

The rise in blood glucose is accompanied with disturbance of ROS production, lipid and hepatic enzyme profiles, making diabetic patients at high risk for diabetic complications including fatty liver degeneration [34]. Similarity, hepatotoxic, nephrotoxic, and abnormality of fatty acids metabolism in adipose tissue are well-documented effects of STZ in experimental animals [20, 35, 36]. In the current work, STZ mice exhibited increased ALT levels, along with other hepatic enzymes, including AST and ALP, but these enzymes were reduced in PILE-pretreated mice. However, it remains to be addressed whether PILE has a direct effect on hepatocytes or an indirect effect on preventing oxidative damage due to STZ toxicity. Another beneficial effect of PILE is to lower ALT enzymes found primarily in the liver, which is normally used to determine liver health. Elevated ALT levels have been previously reported to be one of the deleterious effects of STZ cytotoxicity [37]. According to the research of Yang and Kang (2018), quercetin and resveratrol treatment improved the activities of hepatic glucose metabolic enzymes and serum lipid profiles by mobilizing lipids from muscles in STZ-induced diabetic rats. Noteworthy, the combined treatment of quercetin and resveratrol exhibited the most preventive effect on the diabetic rats [38]. These results provide further evidence that PILE could exert its beneficial effect against STZ cytotoxicity through the presence of quercetin and resveratrol.

Apoptosis is reported to be the main mechanism of β-cell death in both T1DM and T2DM, regardless of their etiology [2, 39]. Previous studies have indicated that the mechanisms leading to cytokine-induced β-cell death (T1DM) and diet-induced progressive β-cell failure (T2DM) share the activation of a final common pathway involving IL-1β, NF-κB, and Fas [4]. Pathologically, the cytotoxic macrophages release β-cell-cytotoxic cytokines, including IFN-γ, TNF-α, IL-1β, and free radicals. Once released, IFN-γ leads to the recruitment and phosphorylation of STAT1. After being phosphorylated, STAT1 homodimerizes and migrates to the nucleus to initiate the transcription of apoptosis-inducing genes. In the meantime, TNF-α activates caspase-8, which in turn activates effector caspase-3. Lastly, upon binding to its receptor, IL-1β activates NFκB pathways, leading to the activation of iNOS which eventually induce β-cell death [1, 4]. On the other hand, the intrinsic pathway is involved in the mitochondria, responding to a deleterious effect of chronic hyperglycemia on β-cells through the release of cytochrome C, which subsequently activates caspases -9 and -3, leading to apoptosis [2, 39]. In the present study, total levels of pancreatic proinflammatory cytokines (IFN-γ, TNF-α, IL-1β), inflammatory-related markers (NFκB, pSTAT1, and iNOS), and apoptotic markers (capspases-3, -8, and -9) were upregulated in untreated STZ mice. Almost of these disturbances were corrected by pretreatment with PILE 100 (Fig 6E). Of note, detection of protein expressions of IL-1β and cleaved caspase-9 at the 8-week time point appears to be produced in the same amount in all treatment groups. The possible explanation for this phenomenon could result from the adaption of mice to the treatment, which makes these biological markers become less important in the pathogenesis and/or responsiveness to the activators. Similar to our results, the inhibitory effect of resveratrol compound on proinflammatory cytokine actions was previously demonstrated by Lee et al. (2009). The authors showed that the exposure of isolated rat pancreatic islets to cytokines lead to numerous an increased DNA binding of NF‐κB, increased production of NO, and expression of iNOS. All these deleterious effects were attenuated by resveratrol [40]. The authors claimed that this protective action of resveratrol against cytokine‐induced dysfunction of β-cells is due to the ability of resveratrol to activate nicotinamide adenine dinucleotide (NAD^+^)‐dependent protein deacetylase Sirt1 [40]. Recently, Roslan et al. (2017) confirmed that administration of quercetin not only decreases blood glucose levels in STZ-nicotinamide-induced diabetic rats thought its ability to induce glucose-stimulated insulin secretion but also strengthen antioxidant enzymes, resulting in the reduction of inflammatory cytokines and STZ-induced cell apoptosis in mouse hearts [41]. Collectively, quercetin and resveratrol could significantly be attributed to the preventive effect of PILE against cytokine-induced β-cell death in our mouse model.

Another major finding in this report is the induction of Ki67 protein detected by immunostaining upon pretreatment with PILE. This phenomenon may be the explanation for the lower blood glucose levels seen in PILE 100 mice. No significant upregulation of Ki67 was detected at the 4-week time point and it is possible that the newly formed β-cell may not be mature enough to produce insulin granules. As the study was extended to 8 weeks, β-cells expressing the Ki67 marker remarkably increased. We do not know at the moment what mechanisms could be involved in the PILE-induced β-cell proliferation in our animal model. Based on accumulating evidence, it is suggested that quercetin has ability to facilitates regeneration of β-cells by stimulating the ductal stem cells to regenerate and differentiate into pancreatic islet cells [33]. The underlying mechanism of PILE-induced cell proliferation needs further investigation. Nevertheless, our results support a growing body of evidence that β-cell replication is still ongoing in the pancreas [42, 43].

In conclusion, we have presented original evidence that PILE pretreatment effectively ameliorated cytokine-induced β-cell injury in STZ mice. Moreover, the possible antidiabetic mechanism of PILE was elucidated in that PILE protected β-cells by inhibiting apoptosis and enhancing cell proliferation. This beneficial effects of PILE could be attributed to, at least in part, the presence of antioxidant compounds, quercetin and resveratrol. Based on our *in vivo* studies, PILE is safe to consume and has inhibitory effect on cytokine-induced β-cell apoptosis in STZ mice.

## Supporting information

S1 File Chemical structures by LC-MS of major phytoconstituents of *Pluchea indica* crude leaf ethanol extracts (PILE). (PDF)

S2 File Preliminary phytochemical screening and list of identified compounds by GC-MS of *Pluchea indica* crude leaf ethanol extracts (PILE). (PDF)

S3 File Supplemental raw data. (XLSX)
