# Supplementary material for: Ethanolic extracts of Pluchea indica (L.) leaf pretreatment attenuates cytokine-induced β-cell apoptosis in multiple low-dose streptozotocin-induced diabetic mice
Source: PLoS One. 2019 Feb 19;14(2):e0212133. doi: 10.1371/journal.pone.0212133 (PMC6380574; doi:10.1371/journal.pone.0212133)
Supplement: S1 File — (PDF) [file pone.0212133.s001.pdf]

Supplementary material

S1 File. Chemical structures by LC-MS of major phytoconstituents of *Pluchea indica* crude leaf ethanol extracts (PILE). These compounds include quercetin**,** 6-hydroxykaempferol 7-glucoside, 3,4-dicaffeoyl-1,5-quinolactone, apigenin 7-(2'',3''-diacetylglucoside), trans-trismethoxy resveratrol-d4, and campesteryl ferulate.

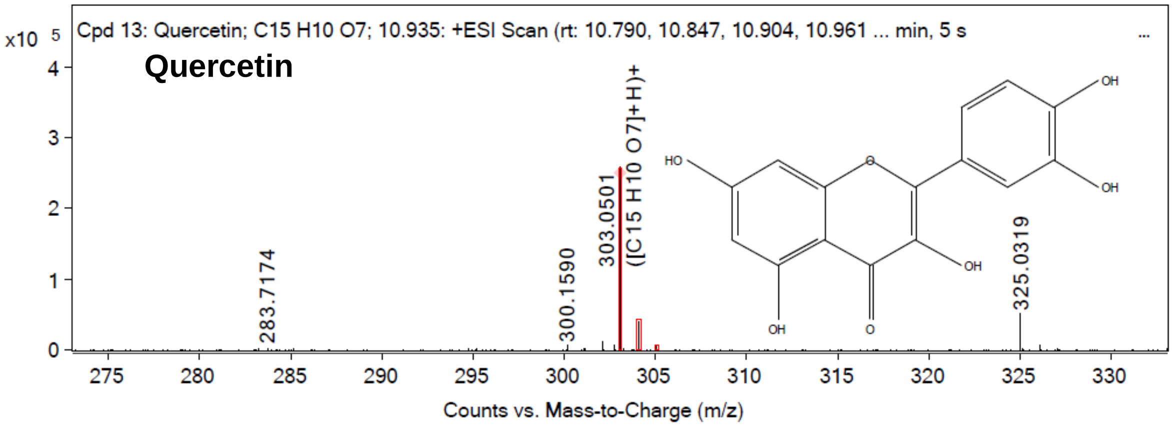

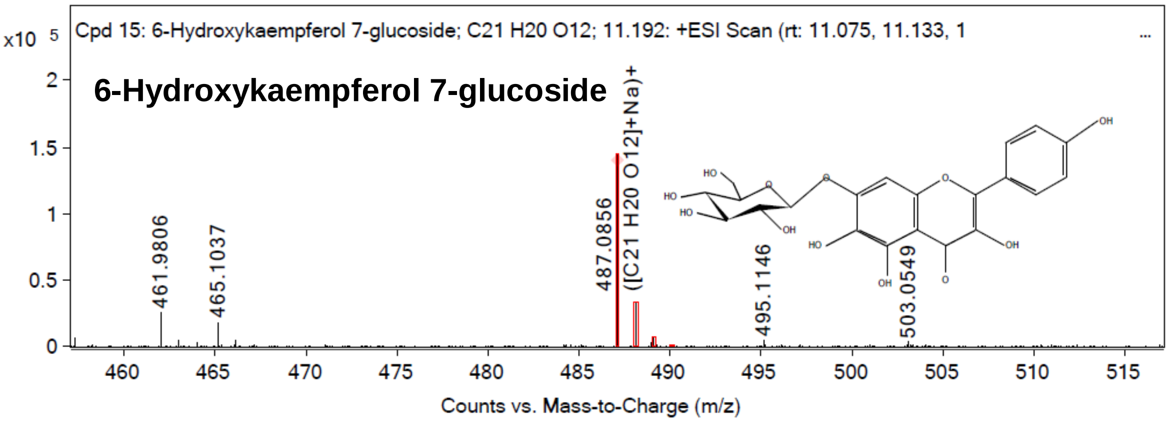

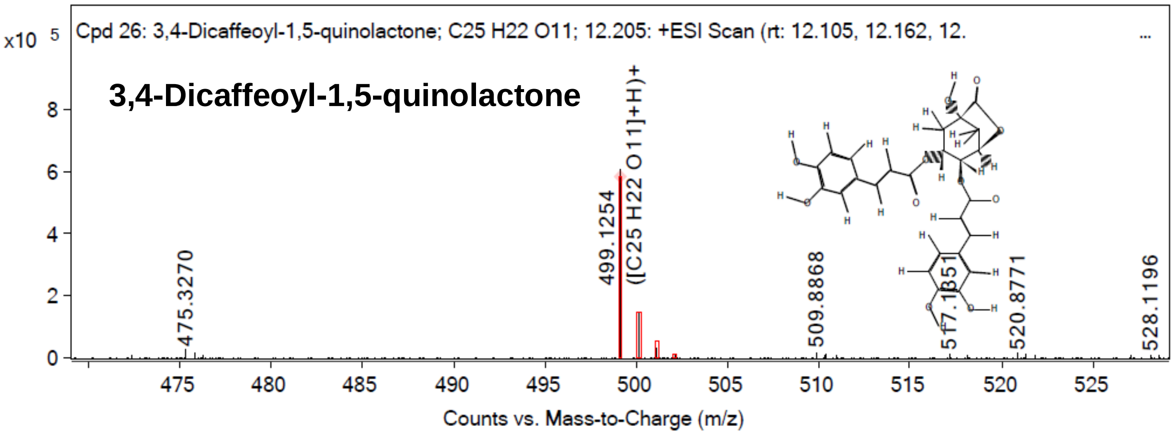

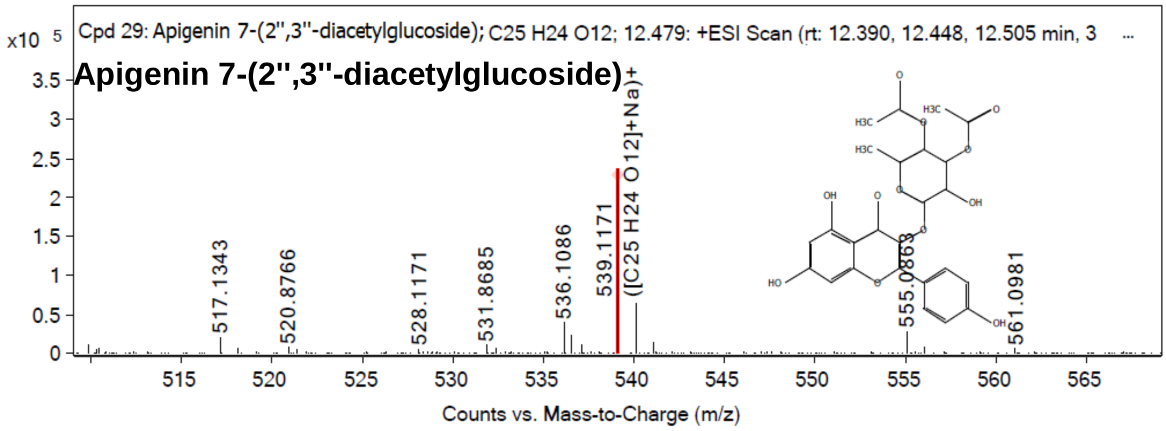
x10 5
Cpd 29: Apigenin 7-(2'',3''-diacetylglucoside); C25 H24 O12; 12.479: +ESI Scan (rt: 12.390, 12.448, 12.505 min, 3 ...
Apigenin 7-(2'',3''-diacetylglucoside)
([C25 H24 O12]+Na)+
517.1343
520.8766
528.1171
531.8685
536.1086
539.1171
555.0863
561.0981
Counts vs. Mass-to-Charge (m/z)

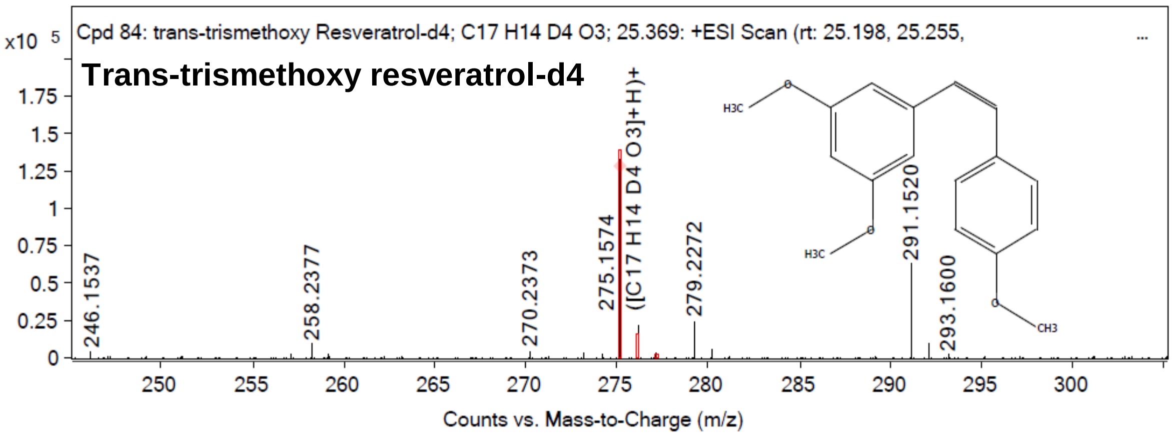
x10 5
Cpd 84: trans-trismethoxy Resveratrol-d4; C17 H14 D4 O3; 25.369: +ESI Scan (rt: 25.198, 25.255, ...
Trans-trismethoxy resveratrol-d4
([C17 H14 D4 O3]+H)+
246.1537
258.2377
270.2373
275.1574
279.2272
291.1520
293.1600
Counts vs. Mass-to-Charge (m/z)

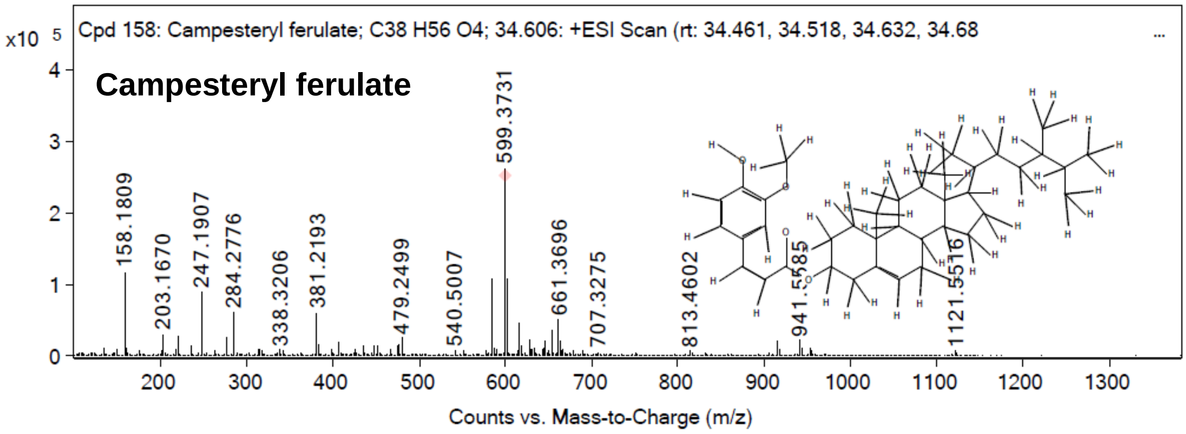
x10 5
Cpd 158: Campesteryl ferulate; C38 H56 O4; 34.606: +ESI Scan (rt: 34.461, 34.518, 34.632, 34.68 ...
Campesteryl ferulate
158.1809
203.1670
247.1907
284.2776
338.3206
381.2193
479.2499
540.5007
599.3731
661.3696
707.3275
813.4602
941.5585
1121.5516
Counts vs. Mass-to-Charge (m/z)
