# Supplementary material for: Ethanolic extracts of Pluchea indica (L.) leaf pretreatment attenuates cytokine-induced β-cell apoptosis in multiple low-dose streptozotocin-induced diabetic mice
Source: PLoS One. 2019 Feb 19;14(2):e0212133. doi: 10.1371/journal.pone.0212133 (PMC6380574; doi:10.1371/journal.pone.0212133)
Supplement: S2 File — (PDF) [file pone.0212133.s002.pdf]

Supplementary material

S2 File. Preliminary phytochemical screening and list of identified compounds by GC-MS of *Pluchea indica* crude leaf ethanol extracts (PILE).

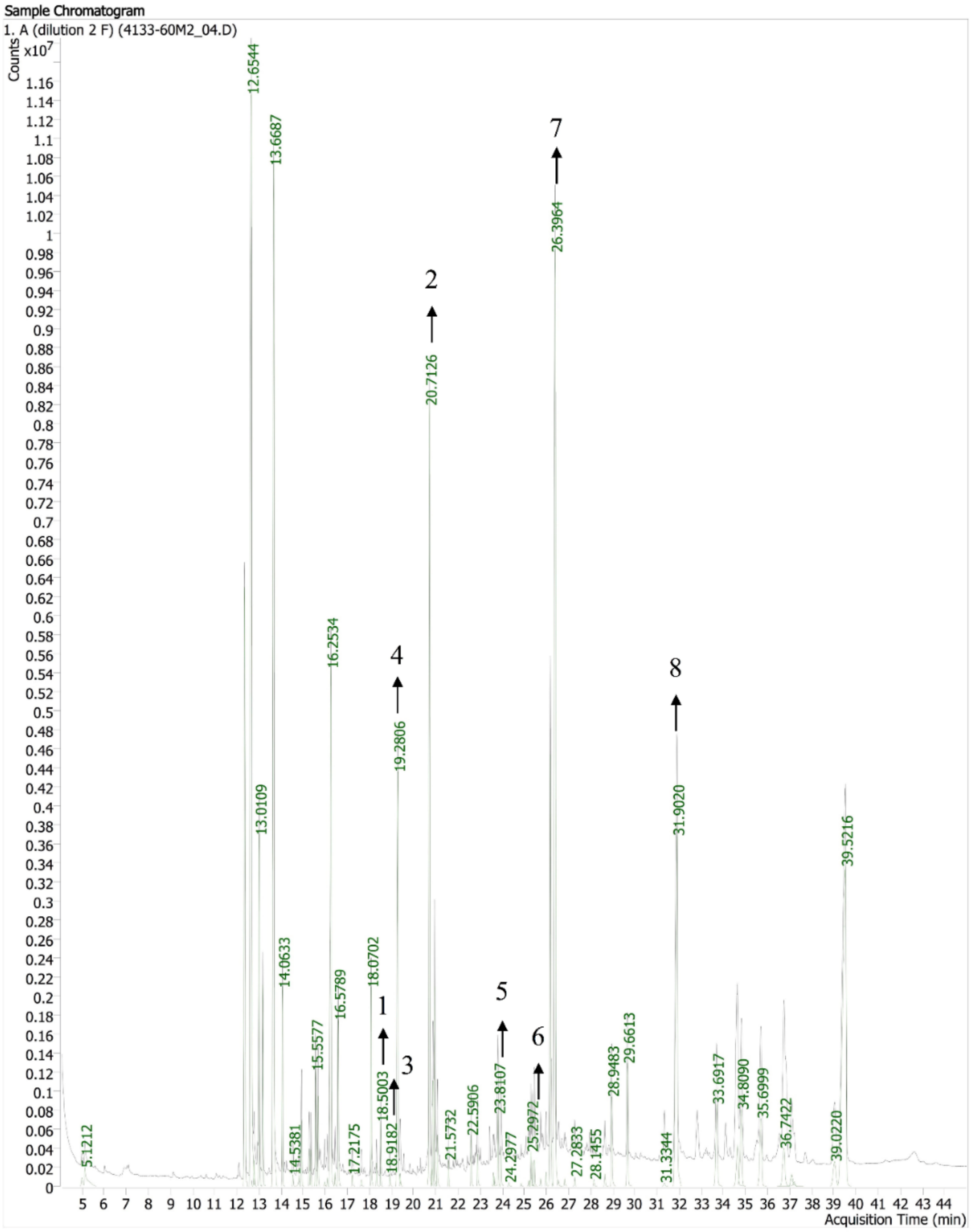

Fig A. **The preliminary phytochemical screening of PILE analyzed by GC-MS.** Mass peaks showed that squalene (10.79%, peak No. 7) is the most abundant component; stigmasta-5,22-dien-3-ol (6.23%, peak No. 8) represented the second largest group, followed by phytol (5.50%, peak No.2) and n-hexadecanoic acid (4.16%, peak No.4).

Table A. List of identified compounds by GC-MS detection of PILE**.**

Peak number	Retention Time (Minute)	Identified compound name	Component Area	%
1	18.5003	2-Hexadecen-1-ol, 3,7,11,15-tetramethyl-, [R- [R*, R*-(E)]]- (Phytol)	1170041.2	0.31
2	20.7126	Phytol	20533197.6	5.5
3	18.9182	5-(Methylthio) piperonal	277907.0	0.1
4	19.2806	n-Hexadecanoic acid	15459950.9	4.16
5	23.9412	Octadecanoic acid, 2-hydroxy-1-(hydroxymethyl)ethyl ester	835204.5	0.2
6	25.3710	11,14,17-eicosa-trien-oic acid, methyl ester	549456.0	0.1
7	26.3964	Squalene	40060668.8	10.79
8	31.9020	Stigmasta-5,22-dien-3-ol	23116033.4	6.23
